# Ferroptosis: A double-edged sword

**DOI:** 10.1038/s41420-024-02037-9

**Published:** 2024-05-30

**Authors:** Shengmei Wang, Qiuyan Guo, Lili Zhou, Xinhua Xia

**Affiliations:** School of Pharmacy, Hunan University of Chinese Medicine, Changsha, Hunan 410208 China

**Keywords:** Cardiovascular diseases, Cancer, Cell biology, Haematological diseases

## Abstract

Ferroptosis represents a form of programmed cell death that is propelled by iron-dependent lipid peroxidation, thereby being distinguished by the prominent features of iron accumulation and lipid peroxidation. Ferroptosis has been implicated in numerous physiological and pathological phenomena, with mounting indications that it holds significant implications for cancer and other medical conditions. On one side, it demonstrates anti-cancer properties by triggering ferroptosis within malignant cells, and on the other hand, it damages normal cells causing other diseases. Therefore, in this paper, we propose to review the paradoxical regulation of ferroptosis in tumors and other diseases. First, we introduce the development history, concept and mechanism of ferroptosis. The second part focuses on the methods of inducing ferroptosis in tumors. The third section emphasizes the utilization of ferroptosis in different medical conditions and strategies to inhibit ferroptosis. The fourth part elucidates the key contradictions in the control of ferroptosis. Finally, potential research avenues in associated domains are suggested.

## Facts

- Ferroptosis is a cellular demise mechanism that relies on the presence of iron, initiated by the buildup of reactive oxygen species (ROS) and lipid peroxidation.
- Ferroptosis has the potential to inflict damage upon normal cells and contribute to the development and advancement of diverse diseases, including cardiovascular disease and neurodegenerative disorders.
- Ferroptosis exerts inhibitory effects on tumor cell proliferation and impedes the migratory and invasive capabilities of tumor cells.

## Open questions

- How does ferroptosis accurately target cancer cells without harming normal cells?
- Can nanotechnology enable precise modulation of ferroptosis?
- What is the ultimate effector of ferroptosis?

## Introduction

In both physiological and pathological contexts, cellular demise represents an inescapable and pivotal event within the life cycle, signifying the conclusion of cellular existence. There are two main categories of cell demise, namely unintentional cell demise and controlled cellular demise. The latter category encompasses various forms such as programmed cell death (apoptosis), self-degradation (autophagy), inflammatory-induced cell death (pyroptosis), and iron-dependent regulated necrosis (ferroptosis). Ferroptosis is a cell death mechanism that relies on the presence of iron and is triggered by the accumulation of reactive oxygen species (ROS) and lipid peroxidation. Additionally, it represents a novel modulable mechanism for cellular death. The fundamental mechanism of ferroptosis involves the generation of harmful free radicals through the Fenton reaction, triggered by divalent iron ions (Fe^2+^). These free radicals then facilitate the peroxidation process of abundantly present polyunsaturated fatty acids on the cellular membrane, ultimately resulting in cell demise. In addition to the presence of Fe^2+^, regulatory factors for ferroptosis include the reduction in glutathione (GSH) levels or the inactivation of glutathione peroxidase 4 (GPX4). GPX4 relies on GSH as a crucial co-factor, and when GSH is absent or GPX4 activity decreases, it can result in lipid peroxidation and cellular damage [1]. In the field of disease treatment, particularly cancer, ferroptosis has gained significant attention in recent years. The use of Sulfasalazine and Erastin as inducers have shown promising results in inhibiting tumor cell proliferation [2]. In addition, the use of nanotechnology to deliver ferroptosis inducers to tumor cells can further increase anti-tumor effects. For instance, Yu et al. encapsulated erastin within FA-labeled exosomes to target triple-negative breast cancer for therapeutic intervention [3]. Therefore, the enhancement of cancer treatment effectiveness can be achieved through the induction of ferroptosis. Nevertheless, it is crucial to note that apart from its potential anti-tumor benefits, ferroptosis has been associated with the onset and progression of various diseases such as cardiovascular disorders and neurodegenerative conditions. For instance, the presence of iron buildup is frequently observed in the brains of patients suffering from neurodegenerative disorders. The excessive levels of free iron can induce oxidative stress, particularly within the brain, where its accumulation in specific regions has been strongly linked to the pathogenesis of various neurodegenerative diseases owing to the relatively limited antioxidant defense capacity exhibited by this organ [4]. The hippocampus, cortex, and other regions of the brain in individuals with Alzheimer’s disease (AD) exhibit an atypical buildup of iron. This dysregulation of iron levels within the brain is responsible for neuronal death, indicating a strong association between ferroptosis and the onset and progression of AD. Hence, comprehending the precise workings of ferroptosis, strategies to trigger ferroptosis in cancerous growths, its potential therapeutic applications, and approaches to restrict ferroptosis are of utmost significance.

**Fig. 1:**
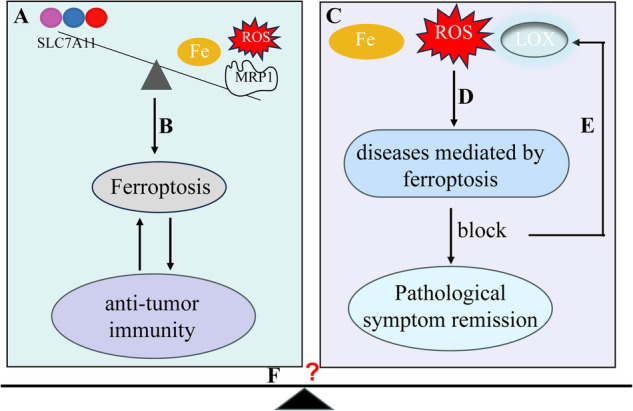
Ferroptosis can be likened to a two-sided blade. **A** The induction of ferroptosis in tumor cells can have anti-tumor effects. **B** Implementing specific strategies to trigger ferroptosis in tumor cells can enhance anti-tumor efficacy. **C**, **D** Excessive occurrence of ferroptosis in normal cells may contribute to disease progression. **E** Inhibiting ferroptosis in normal cells could potentially alleviate the onset of certain diseases. **F** Ferroptosis maintains a delicate balance between tumor cells and normal cells.

In this particular context, we examine the discoveries surrounding ferroptosis and its underlying mechanisms, approaches to trigger ferroptosis in cancerous growths (Fig. 1A, B), the impact of ferroptosis on systemic ailments in humans (Fig. 1C, D), as well as strategies for impeding the occurrence of ferroptosis (Fig. 1E). In addition, we analyzed key contradictions in the application of ferroptosis in tumors versus normal cells (Fig. 1F).

## Overview and discovery of ferroptosis

**Fig. 2:**
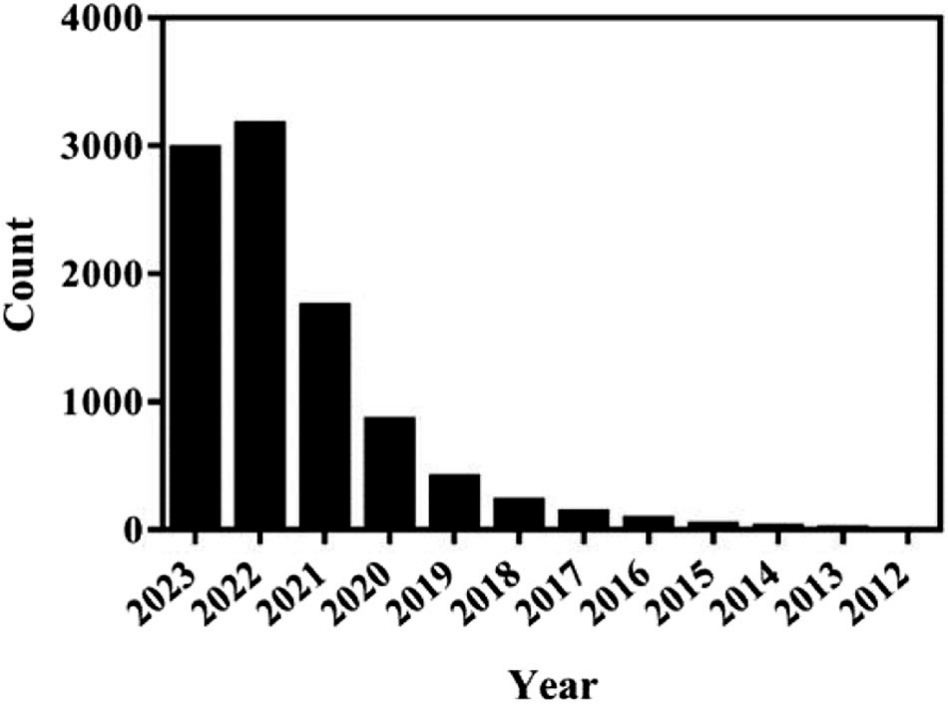
The quantity of research studies on ferroptosis. There have been reports involving ferroptosis in PubMed in the past 12 years.

We summarized the reports involving ferroptosis over the last 12 years through pubmed (Fig. 2). We have observed a strong correlation between the emergence of numerous diseases and the prominence of ferroptosis in recent times, leading to significant interest among researchers. Ferroptosis, a form of cell death triggered by small molecules, is widely recognized as an iron-dependent oxidative process. The occurrence of ferroptosis arises from the disruption in the equilibrium between intracellular ROS production and breakdown. Ferroptosis inducers exert their effects on GPXs, either directly or indirectly, via various pathways. This ultimately leads to a decline in cellular antioxidant capacity, accumulation of ROS, and subsequent oxidative cell death.

**Fig. 3:**
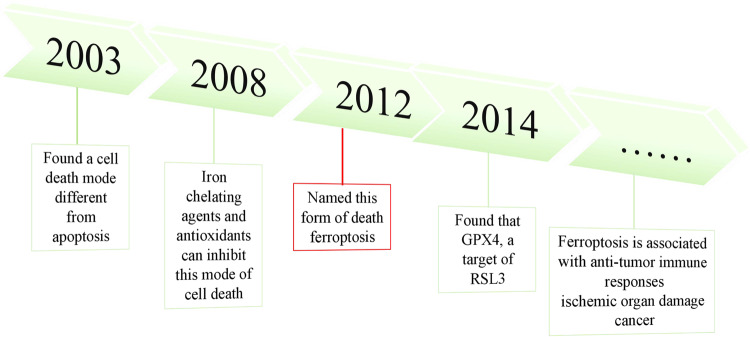
The discovery of ferroptosis. A distinct form of cell death, divergent from apoptosis, was identified in 2003. The defining characteristics of this unique mode of cellular demise were elucidated in 2008. Subsequently named ferroptosis in 2012, the underlying mechanism was unraveled in 2014.

In 2003, Dolma et al. [5]. conducted a study on the impact of erastin, an anti-tumor drug, on tumor cells carrying RAS gene mutation. They identified a distinct mode of cell death that differed from apoptosis; however, it remained unnamed during that period (Fig. 3). In 2008, Yang and Stockwell [6] revealed that ras-selective lethal compound3 (RSL3), induces cell death in a manner resembling erastin. The inhibition of this form of cell death can be achieved through the use of iron chelating agents and antioxidants, indicating its association with iron and ROS. In 2012, Dixon et al. [2]. officially designated this type of demise as ferroptosis due to its distinctive features: a cell death process that relies on iron and is not related to apoptosis, characterized by the accumulation of reactive oxygen species within cells. Mechanistically, erastin inhibits the uptake of cystine through the cystine glutamate transporter receptor (System Xc-) and leads to a depletion of GSH. In a subsequent study conducted by Yang et al. after 2 years, it was discovered that GPX4, which is targeted by RSL3, plays a crucial role in regulating ferroptosis across various types of cancer cells [7]. Receiving extensive recognition in diverse fields of biomedical investigation, such as immune responses against viruses or tumors, organ damage caused by ischemia, and cancer, ferroptosis has garnered significant attention.

## The mechanism of ferroptosis

**Fig. 4:**
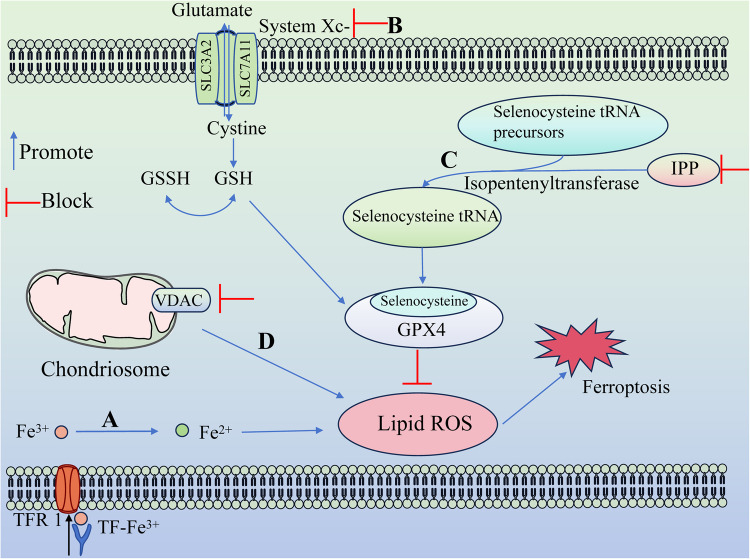
The mechanism of ferroptosis. **A** Iron metabolism. **B** Inhibition of System Xc-. **C** Mevalonate pathway. **D** VDAC channel.

The cellular mechanism of ferroptosis primarily relies on the interplay between two opposing biochemical processes, namely, the production and removal of lipid peroxidation. The process of lipid peroxidation leading to ferroptosis is driven by the utilization of iron and polyunsaturated fatty acids (PUFAs), while the regulation of ferroptosis is reversed by GPX4 using reduced GSH as a substrate. Ferroptosis occurs when cells fail to efficiently eliminate excessive intracellular reactive oxygen species through antioxidant mechanisms, resulting in the accumulation of oxidized lipids. Numerous physiological processes are involved in regulating this mode of death, such as iron metabolism and lipid metabolism (Fig. 4).

### Iron metabolism

The physiological level of iron is strictly regulated through intracellular iron cycling [8]. This process plays a crucial role in oxygen transportation, DNA synthesis, and acts as a coenzyme in the tricar boxylic acid (TCA) cycle and electron transport chain, ultimately impacting ATP production. Research has indicated that the binding of Fe^3+^ to transferrin receptor 1 (TFR1) occurs, followed by its localization in the endosome subsequent to traversing the cell membrane. Within the endosome, Fe^3+^ undergoes reduction to Fe^2+^, which is subsequently released from the endosome into the cytoplasm’s unstable iron pool through divalent metal transporter 1 (DMT1). The surplus iron is stored as ferritin light chain (FTL) and ferritin heavy chain 1 (FTH1), primarily responsible for safeguarding cells against iron toxicity by storing unstable iron in a non-toxic, mineralized form. In situations where intracellular iron ions are excessively abundant, reactive oxygen species (ROS) accumulate via Fenton reaction, thereby promoting ferroptosis. Research has indicated that the application of iron chelators can impede erastin-triggered ferroptosis, while introducing external iron supplements to induce intracellular iron surplus enhances lipid peroxidation and amplifies erastin-induced ferroptosis [9]. Therefore, ferroptosis can be influenced by the regulation of iron ions.

### Lipid ROS metabolism

Lipid ROS metabolism plays a crucial role in the occurrence of ferroptosis. This is primarily attributed to the involvement of System Xc-, GPX4, and voltage-dependent anion channels (VDACs), which exert their functions by modulating lipid ROS metabolism.

1. System Xc- is a cystine/glutamate antiporter protein composed of SLC7A11 (xCT) and SLC3A2 (CD98) [10]. Its function involves importing cystine from outside the cell while simultaneously exporting glutamate out of the cell. Cystine serves as the primary component for cellular production of GSH and plays a crucial role in upholding the equilibrium of redox levels within both intra- and extracellular environments. Repression of System Xc- results in the transcriptional upregulation of SLC7A11 [11], which was observed to be elevated during ferroptosis induced by erastin and sulfasalazine. Furthermore, the downregulation of the SLC7A11 gene using siRNA interference resulted in increased susceptibility of HT-1080 cells to erastin-induced ferroptosis. Conversely, overexpression of the SLC7A11 plasmid in HT-1080 cells enhanced their resistance against ferroptosis [2]. Therefore, the hindrance of System Xc- impedes the uptake of cystine and diminishes the production of GSH, a vital coenzyme for GPXs’ functionality. Consequently, this leads to a decline in GPXs activity, reduced cellular resilience against peroxidation, and accumulation of lipid reactive oxygen species. Ultimately, these factors contribute to cellular ferroptosis.
2. There exist numerous members within the GPXs family, such as GPX1 ~ GPX8 [12], Among them, GPX4 assumes a more significant role in ferroptosis. The direct impact of RSL3 on GPX4 results in the inhibition of its activity. Consequently, this leads to a decrease in the cell’s antioxidant capacity and an accumulation of ROS, ultimately triggering ferroptosis [7]. Additionally, the mevalonate pathway (MVA) has the potential to impact GPX4 through its regulation of selenocysteine tRNA maturation, leading to cellular ferroptosis. Selenocysteine is an essential amino acid found in the active center of GPX4 and requires a specialized transporter known as selenocysteine tRNA for insertion [13]. The maturation process of selenocysteine tRNA relies on isopentenyltransferase’s ability to transfer isopentenyl from isopentenylpyrophosphate (IPP) to precursor molecules of selenocysteine tRNA [14]. Notably, IPP is produced by the MVA pathway. Consequently, modulation of the MVA pathway can influence the synthesis of selenocysteine tRNA by downregulating IPP levels, thereby interfering with GPX4 activity and ultimately resulting in ferroptosis.
3. VDACs are a protein type that can be found extensively throughout the outer membrane of mitochondria, and their primary role is to uphold the permeability of this particular membrane. VDACs consist of three subtypes, specifically VDAC1, VDAC2, and VDAC3. According to the study conducted by Yagoda et al. [15]. the closure of the VDAC2/3 channel by erastin was observed to result in a decrease in the oxidation of nicotinamide adenine dinucleotide hydrogen (NADH). Additionally, there was a reduction in the synthesis of reduced nicotinamide adenine dinucleotide phosphate (NADPH). NADPH supplies hydrogen to convert oxidized glutathione (GSSG) back into its reduced form, glutathione (GSH), leading to a reduction in the overall intracellular content of GSH due to decreased synthesis. In addition, GSH, under the action of GPX, reduces H_2_O_2_ to water, while itself is oxidized to GSSG. Subsequently, under the action of GPX, especially GPX4 [16], GSSG will be reduced to GSH again by providing hydrogen from NADPH generated in the above pathway [17]. GSH functions as an antioxidant and is crucial for cellular protection against oxidative stress. The GSH content can be decreased by the closure of the VDAC2/3 channel through erastin, leading to an increase in cellular susceptibility to reactive oxygen species and ultimately triggering ferroptosis.

## Ferroptosis in cancer

### Ferroptosis as a tumor suppressor event

In the past few years, significant advancements have been achieved in comprehending the significance of ferroptosis in tumor biology and its potential for cancer therapy. Moreover, it has been observed that various signaling pathways associated with cancer play a crucial role in regulating ferroptosis within malignant cells [18]. Ferroptosis plays a role in the function of various tumor suppressors, such as p53 and BRCA1-associated protein 1 (BAP1), which establishes ferroptosis as a natural defense mechanism against cancer development [19, 20]. In various types of cancer including lung [21], breast [22], colorectal [23], liver [24], gastric [25], esophageal squamous cell carcinoma [26] and melanoma [27], the induction of ferroptosis has been shown to inhibit tumor growth. Furthermore, certain cancer types can be targeted therapeutically by exploiting the inherent susceptibility of some cancer cells to ferroptosis due to their unique metabolism, elevated levels of ROS, and specific mutations [28, 29]. In addition, certain cancer cells exhibit a heightened reliance on the ferroptosis defense mechanism in order to endure metabolic and oxidative stress circumstances. Consequently, interfering with these protective measures proves lethal for cancerous cells, while having no impact on healthy cells [30]. Revealed by the most recent data, it can be inferred that certain cancer cases may possess a potential vulnerability that could be targeted through ferroptosis. Ferroptosis is also recognized as a significant cellular demise reaction induced by diverse cancer treatments, encompassing radiation therapy (RT), immunotherapy, chemotherapy, and targeted therapies [31–34].

On the contrary, ferroptosis has been shown to impede the migration, invasion, and metastasis of cancer cells. For instance, PPy@Fe_3_O_4_ nanoparticles have demonstrated the ability to suppress the proliferation and metastasis of colorectal cancer by inducing ferroptosis [35]. SCARA5 influences the proliferation and metastasis of esophageal squamous cell carcinoma by promoting ferroptosis through its interaction with the light chain of ferritin [36]. Additionally, high levels of SLC7A11 expression in HCC are closely linked to lower tumor differentiation, advanced tumor nodular metastasis stage, and unfavorable prognosis [37]. In the same way, neratinib, a strong and permanent inhibitor of pantyrosine kinase, encourages ferroptosis and hinders brain metastasis in a new model of natural human epidermal growth factor receptor 2-positive breast cancer spread [38]. Based on the data provided, it can be inferred that ferroptosis has a suppressive impact on tumor metastasis, suggesting its potential as a tumor suppressor. Therefore, enhancing ferroptosis in cancer cells could offer a promising therapeutic approach.

However, ferroptosis is like a double-edged sword, the promotion of ferroptosis in cancer cells must also take into consideration its potential impact on normal cells and the possibility of side effects. When utilizing ferroptosis for cancer treatment, caution must be exercised to prevent adverse effects on other bodily organs and systems, particularly critical organs such as the cardiovascular system, liver, and kidneys. Furthermore, when employing ferroptosis to modulate the pathophysiology of conditions like stroke, it is essential to conduct further research into its mechanism and potential impacts. This is due to the fact that excessive or inadequate regulation of iron homeostasis can detrimentally affect human health and is closely linked with various metabolic, neurological, and immune-related disorders. Therefore, in addition to focusing on how to promote ferroptosis in cancer cells, there is also a need to focus on how to minimize the damage to normal cells and reduce the risk of potential side effects.

### Methods to promote ferroptosis (Fig. 5)

#### Increased intracellular iron levels

It is widely acknowledged that the induction of abundant ROS in ferroptosis primarily relies on elevating the intracellular iron ion level. Consequently, devising a strategy to enhance intracellular iron accumulation holds promise as an effective approach for antitumor therapy targeting ferroptosis. Normally, intracellular iron cycling processes strictly regulate intracellular iron homeostasis. Extracellular iron can be brought in through the ransferrin (TF) and its carrier protein, the transferrin receptor (TFR). Intracellular iron is stored and transported as ferritin complexes, primarily ferritin (Fn). The export of intracellular iron is regulated by the sole known iron efflux protein in mammals, called ferroportin (FPN), responsible for facilitating the transportation of iron out of cells [39]. In recent years, researchers have developed various strategies targeting the iron transport process to enhance intracellular iron enrichment resulting in increased sensitivity to ferroptosis. (1) Targeting proteins involved in dysregulated iron metabolism. Because TF has a high affinity with the TFR1 membrane receptor, which is often overexpressed in cancer cells, transferrin is an excellent binding partner for chemotherapy. For example, the RAS-RAF-MEK pathway plays a crucial role in promoting ferroptosis in certain cancer cell lines. This could be attributed to the ability of oncogenic RAS to enhance intracellular iron levels through upregulating TFR and downregulating Fn expression [15]. (2) Direct increase in intracellular iron. Nanotechnology is mainly used to construct iron-based nanocarriers to deliver iron intracellularly. Previous studies have predominantly employed solid nanocrystals composed of iron, such as nanoparticles of iron oxide and FePt, to serve as sources of iron for initiating Fenton chemistry within tumors [40, 41]. However, the efficiency of iron release is limited in these solid nanocrystals. As a result, alternative iron-based nanocomposites such as amorphous iron (Fe 0) nanometallic glasses and metal-organic frameworks (MOFs) have been devised to enhance the effectiveness of iron release within tumors [42, 43]. For instance, Zhang et al. developed a nanodelivery system (RF@LA-Fe-MOF) based on Fe (III) to enhance iron levels and induce ROS production for the purpose of initiating ferroptosis as an antitumor strategy. Within the tumor microenvironment, RF@LA-Fe-MOF disintegrated and discharged Fe (III), RSL3, and iFSP1. GSH effectively reduced Fe (III) ions into Fe (II), thereby facilitating the degradation of MOF nanoparticles. Subsequently, Fe (II) ions reacted with H_2_O_2_ to generate ROS and initiate ferroptosis [44]. Furthermore, intracellular iron overload can be induced by certain exogenous substances that contain iron (such as heme chloride, FeCl_2_, and (NH_4_)_2_Fe(SO_4_)_2_), leading to the effective initiation of ferroptosis. Therefore, ferroptosis can be promoted to exert antitumor effects by disrupting intracellular iron homeostasis.

**Fig. 5:**
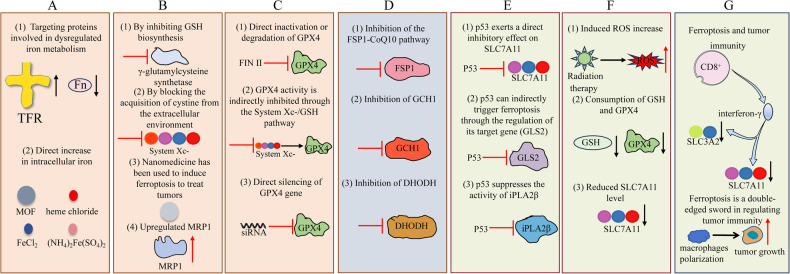
Methods to promote ferroptosis. **A** Increased intracellular iron levels. **B** Increased GSH consumption. **C** Inhibition of GPX4. **D** Inhibition of FSP1, GCH1 and DHODH. **E** p53 enhances ferroptosis. **F** Radiation therapy (RT) and ferroptosis. **G** Ferroptosis and tumor immunity.

#### Increased GSH consumption

The synthesis of glutathione, a primary antioxidant in mammalian cells, involves a two-step process facilitated by intracellular enzymes known as glutamate cysteine ligase (GCL) and glutathione synthetase (GSS). This biosynthesis pathway is influenced by the presence of both cystine and cysteine. Ferroptosis can be induced by the depletion of GSH. (1) By inhibiting GSH biosynthesis. The primary factor restricting the rate of GSH synthesis is the availability of cysteine. Cysteine is produced from its precursor cystine and subsequently forms glutamate-cysteine with glutamate, which depends on the activity of the GCL. For example, buthionine sulfoximine (BSO) promotes ferroptosis through inhibition of γ-glutamylcysteine synthetase [45, 46]. (2) By blocking the acquisition of cystine from the extracellular environment. System Xc- can block the acquisition of cystine by inhibiting System Xc- by exchanging intracellular glutamate for extracellular cystine. For example, the depletion of GSH, accumulation of lipid ROS, and occurrence of ferroptosis are observed when System Xc- is inhibited by erastin [2]. (3) Nanomedicine has been used to induce ferroptosis to treat tumors. Nanotechnology and nanomaterials possess the capability to specifically target the fundamental pathways of ferroptosis, thereby offering promising concepts and designs for their clinical implementation. Revealed in various studies, numerous nanomaterials have demonstrated the capability to induce ferroptosis by depleting GSH. For example, manganese silicate nanobubbles with a high content of arginine have the ability to induce ferroptosis through the depletion of GSH [47]. Fei et al. constructed manganese-doped mesoporous silica nanoparticles loaded with dihydroartemisinin (DHA) that were modified with folate-PEG, which exerted anti-tumor effects by combining GSH depletion and ROS generation to induce ferroptosis [48]. Furthermore, the exceptional capacity of Cu(II) to consume GSH has led to its extensive utilization [49]. For example, Li et al. [50]. developed a nanosheet called Cu-hemin-PEG-LA that specifically targets tumors and can be broken down by acid. In an acidic environment, this nanosheet releases hemoglobin chloride and Cu(II). On one hand, the released Cu(II) reduces the amount of intracellular GSH by converting it into GSSG. This reduction in intracellular GSH content leads to a decrease in GPX4 protein expression, ultimately triggering ferroptosis. On the other hand, the released heme chloride significantly enhances HMOX1 protein expression and causes an overload of Fe^2+^ within cells. The combined effect of reducing GPX4 protein levels and increasing intracellular Fe^2+^ results in elevated lipid ROS content, which induces ferroptosis effectively. (4) Multidrug-resistant protein 1 (MRP1) belongs to the ATP-binding cassette transporter family, specifically involved in exporting specific types of chemotherapy drugs. Recently, MRP1 has emerged as a crucial modulator of intracellular GSH levels, exerting a negative regulatory role. Disrupting MRP1 function leads to a decrease in glutathione efflux from cells. Additionally, increased expression of MRP1 can enhance the sensitivity of cancer cells to ferroptosis inducers that specifically target GSH metabolism [51].

#### Inhibition of GPX4

Refractory cancers can potentially benefit from the inhibition of GPX4, which is considered a promising therapeutic target and prognostic marker in various cancer types. An increasing number of researchers are acknowledging the effectiveness of targeting GPX4 for treating these challenging malignancies. We will introduce some theoretical approaches next. (1) Direct inactivation or degradation of GPX4. For instance, compounds known as class II ferroptosis-inducing agents (FIN) have the ability to directly disable or break down GPX4. In particular, RSL3, which falls under this category of FIN compounds, forms a covalent bond with and deactivates the active site of GPX4 on selenocysteine residues. This direct action effectively hinders their lipid peroxidase activity [7]. The anticancer agent hexamethylamine induces ferroptosis through direct inhibition of GPX4 lipid repair activity [52]. (2) GPX4 activity is indirectly inhibited through the System Xc-/GSH pathway. Sorafenib (SFN) is an ferroptosis inducer that can directly inhibit System Xc-, reduce GSH synthesis and indirectly inactivate GPX4, which can lead to the accumulation of toxic lipid ROS, thereby promoting lipid peroxidation and ultimately inducing ferroptosis [45]. To enhance effectiveness, researchers have directed their attention towards nanotherapeutic drugs as a solution to address the challenges posed by inadequate water solubility and limited specificity of GPX4 inhibitors. Zhu et al. constructed a polydopamine-based nanoplatform (Fe(III)PP@SAS) consisting of trivalent iron and salicylazosulfapyridine to enhance the Fenton reaction and inhibit System Xc-, indirectly inactivate GPX4, and achieve synergistic induction of ferroptosis in cancer cells [53]. Liu et al. developed nanoparticles containing SFN within MIL-101(Fe) to trigger ferroptosis in liver cancer cells by suppressing the activity of System Xc- [54]. In addition, Chen et al. constructed a heparin-encapsulated manganese cluster nanoparticles (Mn12), which indirectly suppressed GPX4 activity by depleting endogenous GSH, resulting in elevated lipid peroxidation levels and ultimately leading to ferroptosis [55]. (3) Direct silencing of GPX4 gene. Small interfering RNAs (siRNAs) are RNA molecules consisting of 21–25 base pairs that have the ability to inhibit the expression of targeted genes. siRNAs are able to specifically target intracellular mRNAs to down-regulate the expression of specific proteins [56, 57]. Thus, ferroptosis can be induced by direct silencing of the GPX4 gene by siRNA. Although siRNA has a clear potency and can silence specific target genes to express specific proteins, siRNA is easily degraded by nucleases in the plasma of the body before reaching the target cell. Moreover, the free siRNA was easily removed by filtration of the glomerular basal membrane with a pore size of 6-10 nm. In addition, siRNA itself has strong hydrophilicity, large molecular weight and electronegativity. Consequently, the penetration of free siRNA through the cellular membrane barrier is challenging [57, 58]. Thus, siRNA delivery needs to be dependent on a variety of vectors for targeted delivery at the tumor site. For example, Zhang et al. constructed iron oxide nanoparticles (IONP) co-delivered targeting GPX4 siRNA and cisplatin to directly inhibit GPX4 expression to enhance ferroptosis [59]. In addition, Huang et al. developed a comprehensive nanoplatform for cancer treatment by disguising the iron-siRNA complex with cancer cell membranes. This innovative approach involved utilizing siRNA to target SLC7A11, which effectively hindered the production of GSH by interrupting intracellular cystine supply. Consequently, this indirect inhibition led to the deactivation of GPX4 and subsequently increased lipid peroxide accumulation, ultimately enhancing ferroptosis induced by iron [60]. Nonsense-mediated mRNA decay (NMD) is a surveillance mechanism that helps minimize gene expression errors by breaking down abnormal mRNA transcripts that contain premature stop signals [61]. The main controllers of NMD are up-frameshift (UPF) proteins and smaug (SMG) proteins. Lee et al. discovered that the direct interaction between SMG9 and GPX4 protein leads to the degradation of GPX4 through a small-scale RNAi screening, thereby facilitating tumor suppression via ferroptosis [62].

#### Inhibition of Ferroptosis suppressor protein 1 (FSP1), GTP cyclohydrolase-1 (GCH1) and dihydroorotate dehydrogenase (DHODH)

In addition to the classical methods for triggering ferroptosis mentioned previously, there are non-classical methods that can also lead to ferroptosis. The discussion will be segmented into three sections. FSP1 is a protein that protects cells from the harmful effects of ferroptosis by promoting its activity with the help of Coenzyme Q10 (CoQ10) [63] The active form of CoQ10, ubiquinol (CoQH2), works to inhibit lipid peroxidation. FSP1 sustains the regeneration of CoQ10 in a NADPH-dependent manner and thwarts ferroptosis by suppressing the proliferation of lipid peroxides [64]. (1) Thus, the induction of ferroptosis can be achieved through inhibition of the FSP1-CoQ10 pathway. For instance, the disruption of FSP1 function, facilitated by specific small molecule inhibitors iFSP1 or genetic deletion, facilitates the promotion of ferroptosis [65]. GCH1 and its metabolic product tetrahydrobiopterin/dihydrobiopterin (BH4/BH2) are essential for inhibiting ferroptosis [66]. The synthesis of BH4 is regulated by GCH1, and the level of GCH1 expression affects cellular sensitivity to ferroptosis [67]. Blocking GCH1 leads to insufficient BH4 production, resulting in the accumulation of peroxides and eventual ferroptosis. On the other hand, increasing GCH1 expression specifically boosts BH4 biosynthesis while decreasing ROS generation [68]. (2) Therefore, the inhibition of GCH1 can potentiate ferroptosis in cellular systems. For example, impeding GCH1/BH4 metabolism promotes erastin-induced ferroptosis by inducing ferritin autophagy [69].DHODH is a crucial enzyme found in the mitochondrial membrane. It facilitates the conversion of dihydroorotate (DHO) to orotate (OA) through oxidation, and also aids in the reduction of CoQ10 to CoQH2 [70, 71]. Recent studies have demonstrated that DHODH plays a significant role in the prevention of ferroptosis by eliminating mitochondrial lipid peroxides through the scavenging of free radicals with the assistance of CoQH2 [30]. (3) Inhibiting DHODH enhances the susceptibility of cancer cells to ferroptosis. For example, Nian Liu developed a prodrug QA-SS-TPP with amphipathic properties that inhibits DHODH by releasing the parent drug QA. This disrupts the anti-ferroptosis system in mitochondria, resulting in significant mitochondrial lipid peroxidation and ferroptosis [72].

#### p53 enhances ferroptosis

The p53 gene plays a crucial role in suppressing tumors, as it has the ability to halt the growth and dissemination of cancer cells by triggering cell cycle arrest, encouraging senescence, and promoting apoptosis [73]. Recent research has indicated that p53 is capable of controlling genes linked to ferroptosis in order to impede the advancement of tumors [74, 75]. (1) The expression of SLC7A11 is directly inhibited by P53, leading to the induction of ferroptosis. The lipid oxidase ALOX12 is a crucial regulator involved in the p53-mediated ferroptosis process. Research has indicated that p53 can indirectly stimulate ALOX12 activity by suppressing the transcription of SLC7A11. Upon activation and release, ALOX12 initiates the oxidation of polyunsaturated fatty acids in the cell membrane, leading to significant ROS stress and ultimately triggering ALOX12-dependent ferroptosis [76]. (2) p53 can also indirectly trigger ferroptosis through the regulation of its target genes [19, 77]. One instance is that P53 has the ability to trigger ferroptosis by upregulating the expression of glutaminase 2 (GLS2), reducing GSH levels, and elevating ROS levels [78]. (3) The calcium independent phospholipase A2β (iPLA2β) plays a critical role in regulating ferroptosis and has the ability to remove lipid peroxides. iPLA2β is recognized as a target gene of p53. Malfunction of iPLA2β activity leads to the buildup of lipid peroxides, thus initiating ferroptosis [79]. Contrarily, p53 is capable of inhibiting dipeptidyl peptidase-4 (DPP4) activity, thereby suppressing ferroptosis [46]. Therefore, p53 has the effect of bidirectional regulation of ferroptosis, and targeting p53-mediated ferroptosis can be a new approach for tumor therapy. Precise intervention against p53-mediated ferroptosis may bring new breakthroughs in tumor treatment and provide patients with more effective treatment options.

#### Radiation therapy (RT) and ferroptosis

RT is a widely used method for treating cancer, which involves the precise administration of ionizing radiation (IR) to eliminate malignant cells. It can directly induce various types of DNA damage, which can lead to cycle arrest, senescence or cell death in different ways, including apoptosis, necrosis, autophagy and mitotic catastrophe [80]. Infrared rays not only cause direct harm to DNA but also target water molecules present in cells, leading to their breakdown and, along with the activation of certain enzymes, promoting the generation of ROS. This molecule plays a crucial role in the buildup of lipid peroxides and triggers ferroptosis. The crucial determinant of ferroptosis lies in the composition of polyunsaturated fatty acids (PUFA) within the cellular membrane. PUFA, being a vulnerable substance, is prone to peroxidation under conditions abundant in iron and ROS, consequently resulting in membrane impairment and cellular demise. The main radiotherapy-mediated ferroptosis pathways are as follows (1) Induced ROS increase. Radiotherapy has the potential to induce lipid peroxidation and ultimately trigger ferroptosis by producing significant levels of ROS. This process leads to the formation of free radicals from PUFAs, which, upon interaction with oxygen through the Fenton reaction, generate lipid hydroperoxides (PUFA-OOH) and enhance the expression of ACSL4, a crucial enzyme involved in lipid peroxidation. Collectively, these mechanisms contribute to the promotion of lipid peroxidation [31]. (2) Consumption of GSH and GPX4. IR consumption leads to a decrease in the levels of GSH, which subsequently reduces the effectiveness and expression of GPX4, thereby triggering ferroptosis [81]. (3) Reduced SLC7A11 level. Radiotherapy can activate ataxic telangiectasia mutant kinase (ATM) to inhibit SLC7A11, thereby inhibiting GSH synthesis [82]. However, previous research has indicated that radiotherapy can stimulate the expression of SLC7A11. This could potentially be a defensive mechanism employed by cells to safeguard against ferroptosis. It is plausible that this process involves the activation of Nrf2 and/or ATF4, both of which are typically triggered by radiotherapy and oversee the transcriptional regulation of SLC7A11. Thus, the regulation of SLC7A11 by radiotherapy appears to be background dependent, such as cell line, radiotherapy dose or duration [83].

The radiosensitivity of tumors is significantly influenced by the involvement of ferroptosis. Tumor cells have the ability to increase the expression of SLC7A11 and GPX4, which helps protect them from ferroptosis induced by radiotherapy. Additionally, when SLC7A11 is upregulated in tumors with KEAP1 mutations, it leads to inhibition of ferroptosis and subsequently causes resistance to radiotherapy [83]. Therefore, targeted inhibition of SLC7A11 or GPX4 can sensitize radiotherapy. In addition, inhibition of the synthesis of PUFA-PLs (e.g., ACSL4 inactivation) inhibits radiotherapy-induced ferroptosis and leads to radioresistance [82]; whereas supplementation of PUFA-PLs (e.g., via nanomaterials) or reduction of the synthesis of MUFA-PLs (e.g., ACSL3 inactivation) promotes radiotherapy-induced ferroptosis and sensitizes radiotherapy. Ferroptosis is an effective way to kill tumor cells by radiotherapy, and radiotherapy-resistant tumor cells are often accompanied by reduced levels of ferroptosis. Hence, the integration of a ferroptosis inducer with radiotherapy is employed to heighten tumor radiosensitivity and consequently amplify the effectiveness of radiotherapy.

Ferroptosis is additionally implicated in the occurrence of complications associated with radiotherapy, and promoting ferroptosis in cancer cells can augment the effectiveness of radiotherapy while exacerbating harm to healthy tissues. For instance, Li et al. and Gong et al. observed that the advancement of radiolucent pulmonary fibrosis was hindered by Ferrostatin-1 (fer-1), a ferroptosis inhibitor, through its ability to suppress lipid peroxidation levels and promote GPX4 expression. In contrast, Erastin, a ferroptosis inducer, enhanced fibroblast differentiation by elevating lipid peroxidation levels and suppressing GPX4 expression. This further aggravated the manifestations of radiolucent lung injury [84–86]. Therefore, ferroptosis is double-sided in the course of radiotherapy, and how to strike a balance between the two is still a problem that needs in-depth consideration and further exploration.

#### Ferroptosis and tumor immunity

The tumor microenvironment (TME) is characterized by low oxygen levels, acidic conditions, inflammation, and suppression of the immune system [87]. TME encompasses the surrounding tissue and cell population of tumor cells, which includes immune cells, blood vessels, and stromal cells. These elements play a critical role in the growth and progression of tumors. Specifically, immune cells are key regulators of tumor growth [88]. Within TME, immune cells have the ability to impact whether ferroptosis occurs in tumor cells through various pathways. For example, CD8 + T cells in the TME promote ferroptosis in tumor cells by releasing interferon-γ (IFN-γ) to reduce SLC3A2 and SLC7A11 expression levels [34]. Additionally, the occurrence of ferroptosis in tumor cells can result in the release of various immunostimulatory signals. These include high mobility group box 1 (HMGB1), calreticulin (CRT), ATP, and phosphatidylethanolamine. These signals play a role in promoting the maturation of dendritic cells, enhancing the efficiency of macrophages’ ability to engulf ferroptotic cells, and further facilitating infiltration of CD8^+^ T cells into tumors. Regulatory T (Treg) is resistant to ferroptosis due to the induction of GPX4, and the specific knockout of GPX4 by Treg cells will lead to ferroptosis of Treg cells [89, 90]. Bone marrow-derived suppressor cells (MDSC) are also resistant to ferroptosis. This resistance may be due to the fact that ferroptosis in MDSC is driven by the p53-heme oxygenase-1 (HMOX1) axis mediated by the n-acylsphingoamidase hydrolase 2 (ASAH2) [91]. Consequently, modulation of ASAH2 activity can promote induction of ferroptosis in MDSC, leading to enhanced activation of cytotoxic CD8^+^ T cells infiltrating tumors and suppression of tumor progression. Moreover, Dai et al. discovered that the polarization of macrophages in TME can be induced by ferroptosis, thereby potentially facilitating tumor growth [92]. This indicates that ferroptosis plays a dual role in regulating tumor immunity. Due to the unique characteristics of hypoxia, acidification, and inflammation within the TME, the antitumor effects of cytotoxic T lymphocytes (CTL), natural killer cells (NK), and dendritic cells (DC) are inhibited. Additionally, TME can enhance the activity of immunosuppressive cells, maintaining an immunosuppressive state within the microenvironment and preventing ferroptosis in tumor cells. Therefore, reversing the immunosuppressive state of TME has the potential to induce ferroptosis in tumors.

## Ferroptosis in other diseases

### Overview

**Fig. 6:**
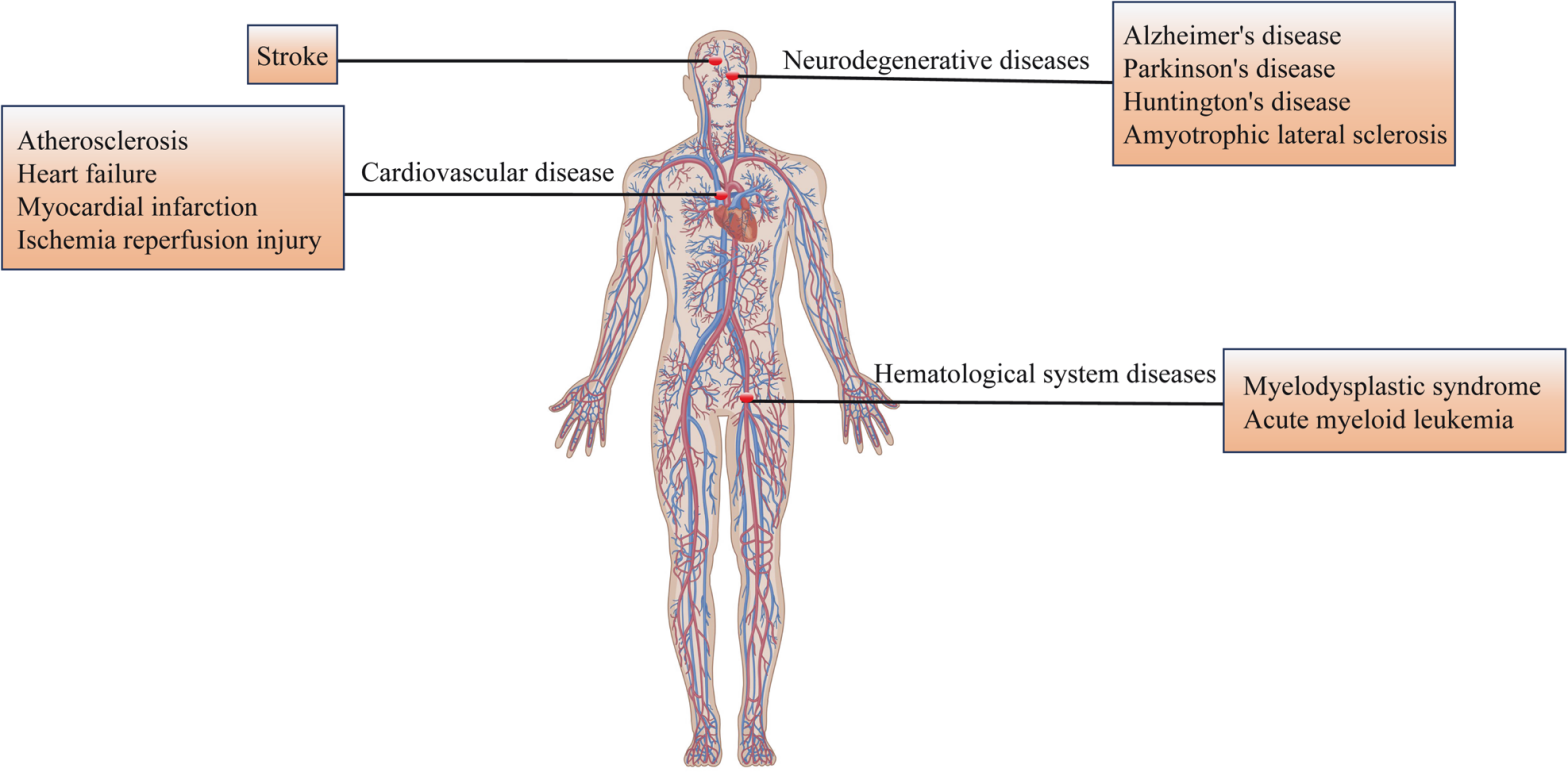
Ferroptosis mediated disease. This figure was drawn using Figdraw (https://www.figdraw.com), export ID: TRPTYb88eb.

**Fig. 7:**
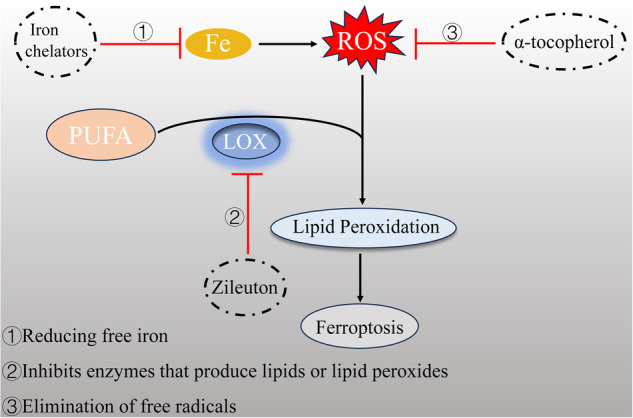
Methods of blocking ferroptosis. (1) Reducing free iron. (2) Inhibits enzymes that produce lipids or lipid peroxides. (3) Elimination of free radicals.

It is a good anti-tumor strategy to induce ferroptosis of tumor cells by specific means. However, due to ROS production and subsequent lipid peroxidation, it can trigger a wide range of pathologies. This has resulted in its connection with the development of various diseases, encompassing those affecting the brain, blood, immune system, and heart. Consequently, gaining a more profound comprehension of how ferroptosis contributes to these diseases and its regulation could unveil fresh possibilities for innovative therapeutic approaches and targets. The following will detail the related diseases mediated by ferroptosis (Fig. 6) and provide effective strategies to inhibit ferroptosis (Fig. 7).

### Diseases associated with ferroptosis-mediated

#### Neurodegenerative diseases

Iron is used for neurotransmitter synthesis, myelin production, myelin sheath formation and neuronal development. Hence, the maintenance of iron balance is crucial for supporting the development and functioning of the brain. However, as individuals age, iron gradually accumulates in the brain, iron homeostasis is disturbed, ROS and free radicals increase significantly, and eventually lead to dysfunction and even death of neurons and glial cells. Ferroptosis has been found to have a correlation with the levels of iron and ROS. Ferroptosis has been linked to neurodegenerative diseases such as Alzheimer’s disease (AD), Parkinson’s disease (PD), Huntington’s disease (HD), and amyotrophic lateral sclerosis (ALS) [93].

1. AD
  AD is a prevalent neurodegenerative disorder that presents with impairments in memory and cognition, as well as reduced language execution capacity. These symptoms significantly impact the elderly’s social interactions and professional performance. The primary pathological characteristics of AD include the presence of age spots resulting from the accumulation of β-amyloid (Aβ) deposits outside cells, neurofibrillary tangles caused by atypical phosphorylation of tau proteins, abnormalities in synaptic function, and loss of neurons [94]. Relevant characteristics linked to ferroptosis, such as disrupted iron metabolism, excessive glutamate activity, and accumulation of lipid ROS, have been observed in the brains of both Alzheimer’s disease patients and mice. The balance of iron levels in Alzheimer’s disease is disrupted due to the accumulation of Aβ and Tau proteins. This leads to an increase in the stability of proteins involved in exporting and transporting iron, which occurs when amyloid precursor protein (APP) is transported to the surface of neurons. Additionally, a decrease in soluble Tau proteins can result in higher levels of iron, while elevated iron caused by Tau also worsens the toxicity associated with Aβ [95]. It can be inferred that the AD process is influenced by iron levels in the brain, and elevated iron levels may serve as a contributing factor to the risk of developing AD. Glutamate excitotoxicity is also an important pathogenetic mechanism in AD, and studies have shown that elevated glutamate in AD patients is associated with abnormal System xc-function, and the abnormalities of glutamate in ferroptosis are also based on abnormal System xc-function [96]. In addition, as a non-negligible trigger in neurodegenerative diseases, oxidative stress has an important connection with AD. Accumulation of Aβ in the AD brain induces an endoplasmic reticulum stress response that decreases GSH levels and leads to increased ROS [97]. Hambright et al. observed that the elimination of GPX4 from neurons within mouse brains led to the deterioration of neurons and impaired cognitive abilities. Nonetheless, administering inhibitors targeting ferroptosis effectively counteracted neuronal degeneration and enhanced cognitive performance in mice [98]. Therefore, it can be hypothesized that ferroptosis is involved in AD pathogenesis.
2. PD
  PD is a prevalent neurodegenerative disorder characterized by slow and progressive onset, and its prevalence ranks second only to Alzheimer’s disease. Clinically, PD is distinguished by movement impairments such as bradykinesia, rigidity, dystonia, and postural instability [99]. The main features of PD are as follows: (1) The most obvious feature of PD patients is the significantly increased content of iron ions in the substantia nigra [100]. The increase of iron ions can cause the increase of hydroxyl radicals and the oxidation of dopamine, resulting in an oxidative environment, which leads to the damage of dopaminergic neurons in the substantia nigra. The abnormal protein of iron uptake and transfer is associated with PD. PD patients have abnormal transferrin, leading to excessive intake of iron ions in nerve cells and resulting in intracellular iron ion accumulation [101]. (2). The presence of Lewy bodies is a significant characteristic of PD, resulting from the aggregation of α-synuclein (α-Syn) in nerve cells to form insoluble substances [102]. This protein is highly expressed in the brain and plays a role in various synaptic processes in neurons. The toxic function of α-Syn is associated with its accumulation, leading to the formation of toxic α⁃Syn oligomers that accumulate in the cytoplasm of nerve cells and disrupt normal nervous system function [103]. In addition, it has been discovered that α-Syn can bind with Fe^2+^ or Fe^3+^ to form α-Syn-iron complexes. Furthermore, α-Syn also functions as a cellular iron reductase, facilitating the conversion of Fe^3+^ to Fe^2+^ [104]. As a result, over-expression of α-Syn leads to elevated levels of iron within the cell. Intranasal administration of the iron-chelating agent deferriamine reduced the formation of pathological α-Syn and demonstrated partial improvement in motor behavior in a rat PD model study [105]. The interaction between iron and α-Syn appears to create a detrimental cycle, and interventions aimed at reducing iron accumulation may effectively mitigate α-Syn buildup and spread. (3) PD is also associated with impaired GPX4 activity. Bellinger et al. found that GPX4 was significantly reduced in SN in patients with PD, suggesting that impaired GPX4 activity may accelerate PD progression [106]. (4) Oxidative stress is a significant characteristic of PD, leading to protein dysfunction, structural changes, DNA oxidation, and cell membrane destruction [107]. It has been found that the oxidative stress observed in PD may be partly due to reduced intracellular GSH levels [108]. In conclusion, there are certain similarities in the pathogenesis of PD patients and the mechanism of ferroptosis. Additionally, in the PD model, anti-ferroptosis drugs also provide protection for dopaminergic neurons against ferroptosis. For example, Chelated iron ions prevent loss and damage of dopaminergic neurons in the substantia nigra compacta by inhibiting ferroptosis, thereby rescuing motor deficits in PD mice [109]. The depletion of GPX4 can be delayed by vitamin E, an inhibitor of ferroptosis, thereby postponing the death of motor neurons [110]. The development of anti-ferroptosis agents presents a significant opportunity for advancing protective therapy in PD patients. Targeting the ferroptosis pathway can effectively reduce neuronal damage, ultimately leading to the delay or mitigation of symptoms and disease progression in PD patients.
3. HD
  HD is a neurological disease caused by genetic factors. Common clinical presentations include chorea, cognitive impairment, and affective disturbances. HD patients exhibit features indicative of ferroptosis, such as decreased levels of GSH, abnormal glutamate signaling, iron accumulation, and lipid peroxidation [111]. Additionally, features indicative of ferroptosis have been observed in animal models of HD. Specifically, there is a blockade in the synthesis of GSH and a decrease in the activity of GPX [112]. These characteristics provide valuable insights into the mechanism of ferroptosis in animals and offer important clues for further research in this area. Recent research has demonstrated that Ferrostatin 1, a ferroptosis inhibitor, effectively suppresses lipid peroxidation and significantly reduces neuronal cell death in isolated brain slices of HD rats [113]. The potential implications of these findings are significant, as they suggest that targeting ferroptosis may be a promising avenue for therapeutic intervention in Huntington’s disease. Furthermore, this research highlights the importance of studying lipid peroxidation and its role in neurodegenerative diseases, shedding light on potential mechanisms underlying neuronal cell death in HD. Overall, these findings provide a foundation for future studies aimed at developing targeted therapies to alleviate the symptoms and slow the progression of HD.
4. ALS

ALS is a degenerative disease of the nervous system that gradually affects specific motor neurons in both the cortex and spinal cord, ultimately resulting in paralysis and fatality. At present, there is a lack of a definitive remedy for ALS and the available treatment choices are restricted. A major obstacle in advancing ALS treatments lies in the uncertainty surrounding the precise mechanism of cellular demise. The presence of excessive iron is a characteristic feature observed in the development of ALS, indicating a potential association with ferroptosis. Golko-Perez et al. detected the presence of iron accumulation within the spinal cord of a mouse model exhibiting symptoms similar to ALS [114]. Reversing the accumulation of iron in the spinal cord of ALS mice can be achieved by employing iron chelating agent. This intervention has the potential to delay the onset of ALS, extend its lifespan, and mitigate motor neuron damage [115]. Furthermore, an elevation in the levels of lipid peroxidation products was observed among individuals diagnosed with ALS, as reported by Devos [116]. Evans et al. conducted a study on mice with GPX4 gene knockout, which resulted in the degeneration and demise of spinal motor neurons. The mice also exhibited an expedited onset of paralysis symptoms resembling those observed in ALS patients [117]. Therefore, targeting the pathways involved in ferroptosis could offer a promising approach for treating ALS. By understanding the molecular mechanisms underlying ferroptosis and its role in neurodegeneration, researchers can develop novel therapeutic strategies to halt or slow down the progression of ALS. Additionally, identifying specific biomarkers associated with ferroptosis in ALS patients could lead to more personalized treatment options and improve patient outcomes.

#### Stroke

Stroke is a disease caused by cell death caused by abnormal blood flow to the brain, and can be categorized into two types: ischemic stroke and hemorrhagic stroke. During hemorrhagic and ischemic strokes, the metabolic equilibrium of iron may become disrupted. On one hand, the upregulation of ferritin, TFR1 and DMT1 facilitates iron influx [118], while on the other hand, FPN inhibits iron efflux [119], leading to excessive intracellular accumulation of iron. This surplus iron catalyzes the Fenton reaction to generate ROS, thereby triggering cell death [120, 121]. In response to this issue, research has demonstrated that administration of the iron chelating agent desferrioxamine (DFO) can mitigate brain edema, neurological impairment and brain atrophy [122]. Additionally, targeting FPN can also suppress ferroptosis and ameliorate neuronal cell death [123]. Furthermore, following a stroke, the Fenton reaction continues to promote lipid peroxidation. Vitamin E has been shown to provide protection against ischemic stroke by inhibiting lipid peroxidation [124]. Additionally, animal experiments have demonstrated that reducing the expression of ACSL4 can effectively inhibit ferroptosis and safeguard mice from cerebral ischemia [125]. While the aforementioned evidence indicates that inhibiting ferroptosis is beneficial in preventing stroke, and certain treatments have shown efficacy, further comprehensive research is necessary to elucidate the potential and feasibility of this type of cell death inhibitor as an innovative therapy for related diseases.

#### Cardiovascular disease (CVD)

CVD poses a significant risk to human health, responsible for approximately one-third of global mortality. This includes various conditions such as atherosclerosis (AS), heart failure (HF), myocardial infarction (MI), and ischemia reperfusion injury [126]. Hence, elucidating the underlying pathophysiological mechanisms contributing to the emergence of cardiovascular diseases holds immense importance in terms of preventing, detecting at an early stage, and providing accurate treatment for CVD. It has been observed that the progression of CVD is associated with disturbances in iron metabolism, accumulation of lipid peroxidation, and excessive ROS levels, which are indicative of ferroptosis [127].

1. AS
  AS is the main cause and pathological basis of various CVD. The pathogenesis of AS is closely related to lipid peroxidation, inflammation, iron metabolism disorder and ROS level increase in related cell types such as vascular smooth muscle cells (VSMC) and endothelial cells [128]. Studies have demonstrated a close correlation between lipid peroxidation and the progression of AS. Firstly, excessive iron accumulation results in the impairment of mitochondria within endothelial cells via pathways involving ROS and cyclooxygenase. This subsequently impacts the inflammatory characteristics of macrophages and facilitates the early development of atherosclerosis [129]. Additionally, iron-induced oxidative stress also contributes to an elevation in inflammatory responses, playing a pivotal role in the pathogenesis of atherosclerosis [130]. Moreover, oxidative stress may impact the structural integrity and flexibility of the vascular wall and participate in both plaque formation and plaque instability [131]. The accumulation of lipid peroxidation is a hallmark of ferroptosis, suggesting that ferroptosis may play a pivotal role in the onset and progression of AS. Mice with AS exhibit endothelial cell damage accompanied by increased ferroptosis, including upregulation of ACSL4 and downregulation of GPX4 [132]. Mice with AS exhibit endothelial cell damage accompanied by increased ferroptosis, including upregulation of ACSL4 and downregulation of GPX4 [133]. These findings suggest that targeting ferroptosis could be a potential strategy for improving AS. Additional investigation into the relationship between ferroptosis and AS may provide valuable insights for the development of novel treatments in the future, potentially benefiting additional patients in improving their health.
2. HF
  HF is commonly observed as the final phase of various cardiovascular diseases, displaying myocardial hypertrophy and fibrosis. However, in certain situations, the development of heart failure can be attributed to cardiomyocyte ferroptosis [134]. The study found that in a mouse model of heart failure induced by the aortic band, lipid hydroperoxide and unstable iron pools were increased in cardiomyocytes. Additionally, the expression levels of GPX4 and ferritin were found to be reduced, indicating that ferroptosis had occurred in cardiomyocytes [135]. With the deepening of research, bioinformatics analysis also provides support for the discovery of related mechanisms. Based on bioinformatics analysis and rat model experiments, CHEN et al. found that knockdown of TLR4 or NADPH oxidase-4 (NOX4) during the development of heart failure could lead to increased expression of GPX4 and FTH1, reduced intracellular unstable iron, decreased lipid peroxidation, inhibition of left ventricular remodeling, and improvement of ventricular function; meanwhile, they also verified that TLR4 is the upstream molecule of NOX4, and proved that the TLR4-NOX4 pathway is one of the pathways related to ferroptosis in the process of heart failure [136]. Furthermore, research has indicated that puerarin possesses the ability to inhibit ferroptosis and enhance cellular viability in cardiomyocytes. Additionally, it can downregulate NOX4 expression while upregulating GPX4 levels, leading to a reduction in ferroptosis and an improvement in HF [135]. All the aforementioned studies have provided evidence for the occurrence of ferroptosis in cardiomyocytes during heart failure progression. Consequently, targeting cellular ferroptosis inhibition could serve as a crucial approach to mitigate HF symptoms.
3. MI
  MI is a cardiovascular disease that poses a significant threat to human health, but its underlying mechanisms are not fully understood. Studies have shown that in mice with MI, reduced levels of ferritin heavy chain 1 (FTH1) at the site of injury lead to increased free iron and oxidative stress, which triggers ferroptosis in cardiomyocytes and ultimately results in cell death and the development of MI [137]. Furthermore, studies have revealed a decrease in the levels of GPX4 protein in MI when compared to healthy tissues. This triggers ferroptosis, ultimately contributing to the occurrence of MI [138]. Liu et al. discovered that resveratrol inhibits ferroptosis by inducing lysine acetyltransferase 5 /GPX4 in myocardial infarction, thereby alleviating myocardial damage [139]. The aforementioned study revealed a correlation between ferroptosis and the onset and progression of myocardial infarction. The inhibition of ferroptosis presents a novel approach for precise management of myocardial infarction.
4. Ischemia reperfusion (I/R) injury

The term I/R injury refers to the process of saving the ischemic heart muscle by restoring cardiac function without improvement or deterioration through early blood flow following procedures such as coronary angioplasty, percutaneous coronary intervention therapy, and coronary artery bypass grafting (CABG) [140]. Accumulation of ROS and excessive iron after reperfusion of ischemic myocardium are significant contributors to myocardial injury and are two main characteristics of ferroptosis. Numerous investigations have shed light on the importance of ferroptosis in causing damage to cardiomyocytes. Chen et al examined lactate dehydrogenase and GPX4 activities and cellular iron, ROS, lipid peroxides, and GSH levels using mouse myocardial ischemia-reperfusion and cultured cardiomyocyte hypoxia/reperfusion models. The myocardial ischemia-reperfusion procedure upregulated embryonic lethal anomalous visual-like protein 1 and cellular iron levels, increased lactate dehydrogenase activity, but significantly decreased GPX4 activity and FTH1 and GSH levels, suggesting that ferroptosis occurs during myocardial ischemia-reperfusion [141]. Tang et al. found that there was no significant change in ferroptosis index (ACSL4, GPX4, iron and malondialdehyde) in myocardial tissue during severe myocardial ischemia. However, with the prolongation of reperfusion time, the levels of ACSL4, iron and malondialdehyde gradually increased, while the levels of GPX4 decreased [142]. Additionally, numerous pharmaceuticals have demonstrated efficacy in mitigating I/R injury through the inhibition of ferroptosis. For instance, baicalein has been shown to effectively suppress ALOX-15 in HT22 cells and ACSL4-mediated ferroptosis in myocardial I/R rats, thereby significantly attenuating myocardial I/R injury [143, 144]. Etomidate has the potential to reduce I/R injury by promoting the activation of Nrf2 and heme oxygenase-1 (HO-1) in order to suppress ferroptosis [145]. Hence, the inhibition of ferroptosis could potentially serve as a promising and efficacious therapeutic approach for I/R injury.

Ferroptosis is a distinct form of cellular programmed demise that significantly contributes to the onset and progression of numerous ailments. The development of CVD is characterized by ferroptosis such as iron metabolism disorders, lipid peroxides accumulation, ROS accumulation, and so on. Controlling ferroptosis has the potential to impact the progression of cardiovascular disease, and targeting this process could be a novel approach for managing CVD. However, the precise impact of ferroptosis on the progression of cardiovascular disease remains uncertain, and its specific mechanism has yet to be fully elucidated. Furthermore, it is important to note that the role and mechanisms of ferroptosis may vary across different types of cardiovascular diseases, necessitating further comprehensive investigations.

#### Hematological system diseases

Red blood cells are high in iron, with 6 g of hemoglobin and 20 mg of iron per 20 mL of blood produced [146]. Thus, hemolysis, hemorrhage, or erythropoietic effects predispose to ferroptosis. During hemolysis, heme mediates the activation and ferroptosis of human platelets through ferritin body regulation [147]. In a mouse transfusion model, the input of long-term stock of red blood cells resulted in increased erythrophagocytosis of red pulp macrophages, causing cell ferroptosis. Hence, an imbalance in iron homeostasis has the potential to trigger a range of hematological disorders [148]. For example, iron generates hydroxyl radicals via the Haber-Weiss reaction and the Fenton reaction, which promote ROS formation, and acute myeloid leukemia (AML) cells induce ferroptosis in AML cells by increasing ROS levels via the HMGB1-regulated RAS-JNK/p38 signaling pathway [149]. In addition, iron overload is often associated with myelodysplastic syndrome (MDS), and iron overload can destroy the bone marrow microenvironment in MDS patients, resulting in decreased bone marrow mononuclear cell viability, decreased GSH and GPX4 activities, and increased ROS. Fer-1 and DFO reverse the growth inhibitory effect of decitabine on MDS cells [150]. Based on the aforementioned data analysis, it can be inferred that the inhibition of ferroptosis in hematological diseases may yield favorable outcomes for patients. This implies that the regulation of iron levels within the body is crucial in mitigating blood system disorders. Therefore, in the management and prevention of blood system diseases, careful consideration should be given to controlling iron intake and metabolism to optimize therapeutic efficacy.

As a part of our research, we have conducted a thorough and extensive analysis on the correlation between ferroptosis and different medical conditions. Our findings indicate that ferroptosis plays an essential role in these diseases. Regulation of ferroptosis-related pathways or protein expression, intracellular ROS levels, and the homeostasis of various metabolic pathways can affect the sensitivity of cells to ferroptosis, thereby inducing ferroptosis. Currently, the investigation of ferroptosis is in its early stages. Exploring the mechanism and significance of ferroptosis in different diseases, as well as suggesting precise therapeutic strategies, holds immense theoretical and practical importance. In the future, more research can be conducted to explore the mechanism of ferroptosis and its role in various diseases, paving the way for targeted treatment strategies. For instance, interventions aimed at modulating intracellular ROS levels or regulating specific metabolic pathways could be explored to mitigate or inhibit the iron death process. Additionally, gene editing technology and alternative approaches may offer potential avenues for altering cellular iron ion handling capacity to reduce its lethal effects.

### Methods of blocking ferroptosis

The pathogenesis of numerous diseases is associated with the accumulation of iron and lipid peroxidation. Consequently, exploring strategies to inhibit ferroptosis could offer a novel approach for preventing and managing disease progression. Three crucial factors contributing to ferroptosis include the availability of intracellular iron, membrane lipid peroxidation, and the loss of antioxidant defenses. At present, inhibiting ferroptosis is mainly divided into (1) reducing free iron, (2) repressing enzymes responsible for synthesizing lipids or generating lipid peroxides, and (3) eliminating free radicals.

#### Reducing free iron

Iron is essential for the implementation of cellular ferroptosis, as indicated by its name. Iron plays a crucial role in facilitating the Fenton reaction, which is responsible for the production of free radicals and the initiation of lipid peroxidation. Moreover, iron is indispensable for activating LOX and POR enzymes that contain iron, responsible for oxidizing membrane PUFA. Furthermore, iron plays a crucial role in the redox metabolic pathways associated with the generation of reactive oxygen species within cells. Consequently, inhibiting the availability of iron becomes imperative to impede ferroptosis. Iron chelators hinder redox reactions and facilitate the effective elimination of iron without any redistribution. Currently, clinically used iron chelators include DFO, deferiprone, deferasirox, and dexpropylenimine [151]. For example, Deferasirox can treat ulcerative colitis (UC) by reducing free iron ions, inhibiting ferroptosis and improving intestinal flora [152].

#### Inhibits enzymes that produce lipids or lipid peroxides

Oxidative stress plays an integral role in causing cellular ferroptosis. Cells in the body have their own antioxidant capacity, thus avoiding oxidative damage; however, when the cell’s own antioxidant capacity is malfunctioning, oxidative damage is triggered, leading to ferroptosis. Therefore, targeting cellular oxidative stress, i.e., inhibiting lipid peroxidation, could be a way to inhibit ferroptosis. For example, Fer-1 can down-regulate prostaglandin endoperoxide synthase 2 expression and up-regulate GPX4 and Nrf2 protein expression. This leads to safeguarding HT-22 cells against oxidative toxicity induced by glutamate and ferroptosis through the inhibition of oxidative stress, as well as reducing the impact of ROS and lipid peroxidation [153]. Zileuton functions as a selective suppressor of 5-LOX, effectively safeguarding ACSL4 overexpressing LNCaP and K562 cells against erastin-induced ferroptosis by impeding the generation of 5-hydroxyeicosatetraenoic acid [154].

#### Elimination of free radicals

Ferroptosis is a pathway in which excessive iron accumulation leads to a burst of intracellular lipid ROS, which in turn causes lipid peroxidation and ultimately cell demise. Reducing or inhibiting ROS can not only block oxidative stress, but also reduce the accumulation of lipid peroxidation end products. For instance, the antioxidant capability of α-tocopherol is demonstrated through its ability to interrupt the chain reaction of auto-oxidation [155].

## Dilemma

In recent years, researchers have carried out thorough studies on the mechanisms of tumor development and found that ferroptosis plays a decisive role in tumor development and treatment, and is now a new strategy for tumor therapy. In addition, ferroptosis was found to have a role in tumor cell proliferation and metastasis. On the one hand, ferroptosis can promote tumor cell apoptosis and inhibit cell proliferation, and on the other hand, it can prevent tumor cell migration and invasion. This means that ferroptosis can be used as a new target for tumor therapy and promote the development of tumor treatment strategies. Some studies have found that ferroptosis can make a contribution to tumor cell death in chemotherapy, radiotherapy and immunotherapy. Therefore, by utilizing the mechanism of ferroptosis, more effective tumor treatment can be developed to improve the therapeutic effect. However, despite the positive effects of ferroptosis on tumor therapy, it is often difficult and risky to apply in practice. Research has shown that ferroptosis can not only be used as a cancer treatment, but also damage normal cells and cause other diseases. How to accurately target cancer cells without harming normal cells in the treatment of tumors is by far the biggest problem, that is, avoiding healthy cell ferroptosis and selectively inducing tumor cell ferroptosis.

Nano-drug delivery system (Nano-DDS) developed using bio-nanotechnology has the unique advantage of improving drug availability and targeted drug delivery. Active targeted delivery and site-specific therapeutics can be achieved by specific surface modifications of the nanodelivery system [156]. On the one hand, its application to ferroptosis therapy can avoid ferroptosis induced disease in healthy normal cells, and on the other hand, precise targeting of tumor cells can improve drug utilization to selectively induce ferroptosis in tumor cells to exert anti-tumor effects. For example, Huo et al. developed a polyethylene glycolized single-atom iron-containing nanocatalyst, which can disperse iron atoms into carbon nanomaterials, and modified polyethylene glycol on the surface of nanoparticles, so that H_2_O_2_ can be easily absorbed and dissociated by amorphous iron, and polyethylene glycolized single-atom iron-containing nanocatalysts can initiate the Fenton reaction under the acidic microenvironment of tumors and single-atom nanocatalysts are well biodegraded and biocompatible without any significant toxic reactions [157]. In addition, nanodelivery systems that combine ferroptosis with other therapeutic approaches may be more effective for cancer treatment. For instance, Zhang et al. developed a bionic nanoparticle composed of ferric teoxide magnetic material. This nanoparticle was designed to simultaneously carry a transforming growth factor-β inhibitor and programmed cell death receptor 1 antibody. The purpose was twofold: firstly, the growth factor-β inhibitor aimed to create an immunogenic microenvironment and elevate H_2_O_2_ levels; consequently promoting iron ions’ involvement in the Fenton reaction to generate hydroxyl free radicals that induce tumor cell death. Secondly, the deceased cells released tumor antigens which further enhance the immunogenetic properties of the microenvironment [158]. However, there are still many problems with Nano-DDS based on ferroptosis: (1) the composition of Nano-DDS based on ferroptosis is complex, and the potential adverse reactions are difficult to control; (2) the structure of Nano-DDS is precise, and the industrial production of the drug is difficult; (3) the cost of treatment is expensive. Therefore, it is of great importance to construct a simple mass-produced nanoparticle that can selectively induce ferroptosis by avoiding ferroptosis in normal cells.

Molecular targeted therapy involves the use of drugs or other substances to specifically target molecules (molecular targets) in order to inhibit the growth and spread of cancer cells, achieving greater efficacy against cancer cells while minimizing damage to normal cells. This field encompasses various areas including small molecule inhibitors, monoclonal antibodies, therapeutic cancer vaccines, and gene therapy [159]. When it comes to ferroptosis, the utilization of molecular targeted therapy in targeting ferroptosis is anticipated to yield more precise and efficacious therapeutic outcomes. Small molecule inhibitors are a class of relatively low molecular weight compounds, typically <900 Da. These small molecule inhibitors can penetrate the cell membrane and enter the cell interior, and can target specific proteins or inactivate kinases, thus disrupting the dysregulated signaling pathway in the carcinogenic process [160]. Dysregulation of protein kinases may lead to abnormal cell growth and thus affect human health [161]. Small molecule inhibitors competitively bind to the active or inactive adenosine triphosphate (ATP) binding sites of tyrosine kinases, thereby exerting a direct impact on tumor cells [162]. Hence, the utilization of small molecule inhibitors to disrupt ferroptosis is anticipated to emerge as a pivotal milestone in the realm of cancer treatment in the foreseeable future. Monoclonal antibodies are capable of targeting specific proteins and exerting inhibitory effects on tumor growth through both direct and indirect mechanisms. In addition to disrupting receptor-ligand interactions, they can also induce apoptosis, impede angiogenesis, and activate immune cells [163]. The monoclonal antibody Nivolumab stimulates a T-cell-mediated immune response by blocking PD-L1 binding to T cells [164]. Recent research has demonstrated that monoclonal antibodies targeting LGR4 can specifically induce ferroptosis and overcome drug resistance in colorectal cancer by inhibiting the LGR4-WNT signaling pathway [165]. The investigation of tumor vaccines represents a significant advancement in the realm of cancer therapy. In the context of the peptid-major histocompatibility complex (MHC), cancer vaccines can stimulate T cells to target specific tumor-associated antigens (TAA), thereby inducing an anti-tumor immune response [166]. Studies have shown that Shichuan Hu et al. constructed fibroblast activating protein-alpha (FAP) genetically engineered tumor cell-derived exosome-like vesicle vaccine (eNVs-FAP) inhibits tumor growth by reprogramming the tumor microenvironment and promoting tumor ferroptosis [167]. Gene therapy aims to disrupt the proliferation and metastasis of cancer cells through the introduction of genetic material, such as DNA or RNA. Specifically, it can restore the normal function of mutated tumor suppressor genes by replacing them, thereby impeding tumor progression. Additionally, genetic material such as siRNA or antisense oligonucleotides can be utilized to inhibit the expression of oncogenes, thereby promoting immune responses and disrupting tumor-related angiogenesis processes [168]. For instance, co-delivery of PD-L1 siRNAs with oral sorafenib has been shown to induce adaptive immune responses and ferroptosis in hepatocellular carcinoma [169]. The development of molecularly targeted therapeutic drugs for ferroptosis may emerge as a crucial direction in the field of cancer therapy in the future. This therapy is anticipated to minimize harm to healthy cells and enhance patients’ ability to withstand tumor treatment. However, molecular targeted therapy must function by inhibiting specific biomarkers essential for cancer progression, making the treatment effective only for patients with tumors expressing these specific biomarkers. Moreover, due to the high clonal and genetic heterogeneity within the tumor, as well as the complex cell signaling network, molecular targeted therapy may lead to drug resistance issues. Therefore, it is crucial to search for prognostic markers that can predict intra-tumor heterogeneity and identify new therapeutic targets in order to enhance the efficacy of molecular-targeted therapy. In addition to the aforementioned factors, there are other variables that impact the efficacy of molecular targeted therapy which must be taken into account. For instance, careful consideration is also required in the selection of appropriate drug dosage and combination regimen to prevent adverse reactions or drug resistance. In conclusion, for a more effective utilization of molecular targeted therapy in ferroptosis for combating cancer and reducing potential drug resistance, it is essential to enhance the comprehensive exploration and comprehension of the mechanisms underlying tumor heterogeneity, novel prognostic markers, and potential therapeutic targets.

Therefore, to achieve precise regulation and promotion of tumor ferroptosis, a deep understanding of the metabolic mechanism and regulatory pathway of tumor cells to ferroptosis is essential. Additionally, nanotechnology can be utilized for targeted delivery of specific anti-tumor drugs or direct delivery of iron ions into cells to enhance intracellular iron levels for precise targeting of tumor cells. In clinical practice, traditional treatment methods such as radiotherapy and chemotherapy can also be combined, while strengthening the design of individualized treatment plans to improve cure rates and survival rates for different patients. Furthermore, molecular targeted therapy, gene editing, and other approaches can be utilized to modulate ferroptosis-related genes and iron ion levels in tumor cells, thereby selectively inducing ferroptosis in tumor cells. In summary, guided by the principles of precision medicine, the ongoing exploration and application of novel technologies and methods to promote tumor ferroptosis will emerge as a crucial direction for future advancements in cancer treatment.

## Conclusion and Perspectives

Ferroptosis is a form of cellular demise triggered by lipid peroxidation that relies on iron and results in excessive production of ROS. With the deepening of research, it was found that on the one hand, anti-tumor effects can be exerted by inducing ferroptosis in tumor cells, and on the other hand, normal cells can be damaged to cause the development of other diseases. Based on this paradox, this paper systematically presents the discovery and fundamental mechanisms underlying ferroptosis, explores its potential applications in disease management, and elucidates strategies for inducing or inhibiting ferroptosis. The aim is to provide enhanced insights into the pertinent utilization of ferroptosis for therapeutic interventions.

The developmental mechanisms of ferroptosis are becoming increasingly clear, and targeted regulation of ferroptosis has theoretical feasibility in the clinical treatment of multisystem diseases. However, there are both opportunities and challenges for the clinical application of ferroptosis regulation. The following are some areas where existing research has limitations but could still be explored in depth: (1) Currently, there are no specific biomarkers available for the identification of ferroptosis. Iron accumulation and lipid peroxidation are commonly observed phenomena in the manifestation of ferroptosis; however, they do not act as the ultimate effectors driving this intricate process. It is important to note that not all damage resulting from lipid peroxidation leads to ferroptosis. Furthermore, the key regulator responsible for orchestrating ferroptosis also plays an active role in regulating cell death mechanisms in other cellular contexts. Therefore, it becomes imperative to explore and identify distinct molecular markers specifically associated with ferroptosis in future research endeavors. (2) Ferroptosis that precisely targets tumor cells without harming normal cells is also by far the paradox. Ferroptosis is like a double-edged sword, which can both exert anti-tumor effects and cause diseases. In the context of ferroptosis, nanotechnology has been utilized by scientists in recent times to alleviate the adverse impacts caused by toxicity. However, challenges such as the industrial-scale production of nano drug delivery systems have hindered its clinical application. Addressing these issues and exploring avenues for integrating nanotechnology with ferroptosis for disease modulation are pressing concerns that require immediate attention. (3) Understanding what the damage threshold is required to induce ferroptosis may also be the next problem to be solved. Iron and ROS play crucial roles in regulating cell proliferation and signaling pathways. By determining the threshold levels, we can effectively manipulate the occurrence of ferroptosis by precisely controlling iron and ROS concentrations in both normal cells and tumor cells.

In addition, drug development ideas and strategies for ferroptosis need to be considered comprehensively. Firstly, we can explore compounds at the molecular level that disrupt the homeostasis of iron ions in cells to prevent normal cell ferroptosis. Secondly, drug design can incorporate modern biotechnology such as protein engineering and gene editing to develop targeted drugs. Additionally, potential anti-ferroptosis active ingredients can be identified through large-scale screening of compound libraries or natural product libraries, with further optimization of their structure and activity. Prior to clinical application, it is essential to thoroughly assess the toxic and side effects of drugs, metabolic pathways, and in vivo dynamics to ensure safe and effective clinical use of new drugs.
